# The Antimicrobial and Antibiofilm Activity of Oregano Essential Oil against *Enterococcus faecalis* and Its Application in Chicken Breast

**DOI:** 10.3390/foods11152296

**Published:** 2022-08-01

**Authors:** Xiangjun Zhan, Yingzhu Tan, Yingmei Lv, Jianing Fang, Yuanjian Zhou, Xing Gao, Huimin Zhu, Chao Shi

**Affiliations:** College of Food Science and Engineering, Northwest A&F University, Yangling 712100, China; zxj666@nwafu.edu.cn (X.Z.); tanyingzhu@nwafu.edu.cn (Y.T.); yingmei@nwafu.edu.cn (Y.L.); 1261102164@nwafu.edu.cn (J.F.); zhouyuanjian@nwafu.edu.cn (Y.Z.); gaoxing123@nwafu.edu.cn (X.G.); xnspxyzhm@nwafu.edu.cn (H.Z.)

**Keywords:** oregano essential oil, *Enterococcus faecalis*, biofilm, chicken breast, flow cytometry, reactive oxygen species

## Abstract

Oregano essential oil (OEO) possesses anti-inflammatory, antioxidant, and cancer-suppressive properties. *Enterococcus faecalis* is a foodborne opportunistic pathogen that can be found in nature and the food processing industry. The goal of this investigation was to explore the antimicrobial action and mechanism of OEO against *E. faecalis*, inactivation action of OEO on *E. faecalis* in mature biofilms, and its application in chicken breast. The minimum inhibitory concentration (MIC) of OEO against *E. faecalis* strains (ATCC 29212 and nine isolates) ranged from 0.25 to 0.50 μL/mL. OEO therapy reduced intracellular adenosine triphosphate (ATP) levels, caused cell membrane hyperpolarization, increased the intracellular reactive oxygen species (ROS), and elevated extracellular malondialdehyde (MDA) concentrations. Furthermore, OEO treatment diminished cell membrane integrity and caused morphological alterations in the cells. In biofilms on stainless-steel, OEO showed effective inactivation activity against *E. faecalis*. OEO reduced the number of viable cells, cell viability and exopolysaccharides in the biofilm, as well as destroying its structure. Application of OEO on chicken breast results in a considerable reduction in *E. faecalis* counts and pH values, in comparison to control samples. These findings suggest that OEO could be utilized as a natural antibacterial preservative and could effectively control *E. faecalis* in food manufacturing.

## 1. Introduction

*Enterococcus faecalis* is a gram-positive, facultatively anaerobic foodborne opportunistic pathogen that is found in both people and animals as commensals [1]. *E. faecalis*, which is widely distributed in soil water and adhering to plant surfaces, can spread through feces and harm the environment [2]. *E. faecalis* commonly contaminates animal and plant food raw materials, such as milk, meat, and vegetables, through the aforementioned medium [3]. Through these contaminated foods, *E. faecalis* can cause nausea, vomiting, diarrhea, abdominal discomfort, dizziness, endocarditis, urinary tract infections, pelvic infections, and other diseases [4]. The overall detection rate of seven species of *Enterococcus* found in foodstuffs retailed (including pork, leafy vegetables, chicken, beef, etc.) in South Korea was 68.2%, and the bacteria were most prevalent in refrigerated chicken (84.1%), and *E. faecalis* was the most frequently identified species in these foods of animal origin [5].

Biofilm has recently become a worldwide public health issue due to the difficulties of equipment damage, food pollution, and human diseases caused by it [6]. Furthermore, biofilm bacterial cells are harder to remove than planktonic bacteria because they are protected by extracellular polysaccharides [7]. Many *Enterococcus* strains can build biofilms on food contact surfaces, such as stainless-steel surfaces, which improve bacteria’s resistance to environmental stress [8,9]. What is worse, most biofilm-producing bacteria like *E. faecalis* are also becoming resistant to chemical reagents such as sodium hypochlorite, hydrogen peroxide, and so on. As a result, natural alternatives for preventing biofilm formation on foods and food-contact surfaces have sparked interest [10].

In recent years, many researchers have focused on plant essential oils, which are acquired from plants by distillation, fermentation, crushing, extraction, hydrolysis, and airing, and the most common method is steam distillation [11]. Oregano essential oil (OEO) is composed of phenolic and alcohol compounds and is used as a food additive approved by the Food and Drug Administration (FDA) because of its natural and safe properties. Furthermore, recent research indicates that OEO is an antioxidant, anti-inflammatory, antidiabetic, and cancer-suppressing agent [12]. OEO also exhibits antimicrobial activity against *Alicyclobacillus* spp. and other microorganisms [13]. The antibacterial activity of OEO against *E. faecalis* has been reported in the previous study [14,15,16]. However, the antibacterial mechanism and antibiofilm of OEO against *E. faecalis* have not yet been clear.

The goal of this investigation was to explore the antimicrobial and antibiofilm activity of OEO on *E. faecalis*, and to assess the antimicrobial activity of OEO against *E. faecalis* in chicken breast meat and its application in chicken preservation.

## 2. Materials and Methods

### 2.1. Reagents

OEO (density 0.939 g/mL at 25 °C, CAS 8007-11-2) and dimethyl sulfoxide (DMSO; analytical grade) was acquired from Sigma Aldrich (Shanghai, China). Brain Heart Infusion (BHI) broth and BHI agar were acquired from Land Bridge Technology Co. (Beijing, China). The rest of the compounds were of analytical grade. Before each assay, OEO was dissolved in DMSO and vortexed for 30 s at room temperature. In a pre-experiment, we investigated the effect of DMSO on *E. faecalis*. The results showed that 0.5% (*v*/*v*) DMSO had no influence on *E. faecalis*. In this experiment, the final concentration of DMSO in all of the sample solutions (treatment and control samples) was 0.1%, which has no apparent effect on the growth of *E. faecalis*.

### 2.2. Bacterial Strains and Growth Conditions

A total of ten *E. faecalis* strains were used in this study. *E. faecalis* ATCC 29212 was acquired from American Type Culture Collection (ATCC, Manassas, VA, USA). Other nine *E. faecalis* strains (Table 1) were originally isolated from the chicken cloacal swab, swine nasal swab, bovine breast swab, and raw milk. In MIC and MBC testing, all ten *E. faecalis* strains were employed, while *E. faecalis* ATCC 29212 was used in all subsequent investigations. For the use in assays, *E. faecalis* strains stored were streaked on BHI agar and cultivated for 24 h at 37 °C. After that, the colony was inoculated into 30 mL BHI broth and cultured at 37 °C for 16 h.

### 2.3. MIC and MBC Determinations

With slight modification, the MICs of OEO against the various *E. faecalis* strains were evaluated using the broth microdilution method advocated by the Clinical and Laboratory Standards Institute [17]. The organisms were diluted in BHI broth to a concentration of 5 × 10^5^ CFU/mL. OEO was added to each well to achieve final concentrations of 2, 1, 0.5, 0.25, 0.125, 0.0625, and 0 (control) μL/mL. BHI broth containing 0.1% DMSO was used as the background sample. A microtiter plate reader (Model 680; Bio-Rad, Saint Louis, USA) was used to measure the optical density (OD) of each well at 600 nm (OD_600 nm_) before and after incubation for 24 h at 37 °C. The MIC was established as the lowest concentration that resulted in a reduction in OD_600 nm_ ≤ 0.05 after 24 h. For MBC determination, 100 μL equivalent volumes suspensions from each well that demonstrated inhibition were distributed on a BHI agar plate. The MBC value was defined as the lowest antimicrobial concentration that prevented bacterial growth after 48 h incubation at 37 °C.

### 2.4. Growth Curves

With slight adjustments, the growth curves were built by using the method given in Shi et al. [18]. Briefly, well-cultured *E. faecalis* ATCC 29212 suspension was diluted to approximately 2 × 10^6^ CFU/mL in BHI broth, whereupon 125 μL culture and equivalent OEO with different concentrations were added into each well in a 96-well plate, and the final concentrations were 2 MIC, MIC, 1/2 MIC, 1/4 MIC, 1/8 MIC, 1/16 MIC, 1/32 MIC, and 0 (control). The 96-well plates were further cultured at 37 °C for 24 h, and cell growth was measured using a microtiter plate reader (Model 680) at 600 nm and 1 h intervals.

### 2.5. Inactivation Effect of OEO against E. faecalis in BHI Broth and PBS

The concentration of *E. faecalis* ATCC 29212 was diluted to 2 × 10^8^ CFU/mL. BHI broth and OEO were mixed to obtain a concentration of 0 (control), 1/2 MIC, MIC, 3/2 MIC, and 2 MIC and then an equal amount of *E. faecalis* suspension was added to each group. The obtained samples were continuously diluted after incubation at 37 °C for 0, 0.5, 1, 2, 4, 6, and 8 h and then inoculated on BHI agar. After incubation at 37 °C for 24 h, the number of *E. faecalis* colonies was counted. Three independent repetitions were performed for each experiment. Phosphate-buffered saline (PBS, pH 7.2) and OEO were mixed to obtain a concentration of 0 (control), 1/2 MIC, MIC, 3/2 MIC, and 2 MIC, and the other steps are the same as above.

### 2.6. Mechanism of Antibacterial Effects

#### 2.6.1. Membrane Potential 

With minor modifications, the approach provided by Hayat et al. [19] was utilized to evaluate membrane potentials of *E. faecalis* ATCC 29212. Briefly, *E. faecalis* bacterial suspension was prepared as described in 2.2 and diluted to 2 × 10^8^ CFU/mL. After centrifugation (8000× *g*, 5 min, 4 °C), the suspension was washed twice with PBS. Then, for 30 min at 37 °C, 125 μL of cell suspensions and 1 μM of the membrane potential-sensitive fluorescent probe bis-(1,3-dibutylbarbituric acid) trimethine oxonol (DiBAC_4_(3); Molecular Probes, Sigma, Louis, USA) were placed in black, opaque 96-well microtiter plates. After that, the mixture was incubated for 30 min at 37 °C before adding four titers of OEO (0 (control), 1/4 MIC, 1/2 MIC, and MIC). After the excitation and emission wavelengths of 492 and 515 nm, fluorescence was measured using a fluorescence microplate reader (Infinite™ M200 PRO; Tecan, Männedorf, Switzerland). 

#### 2.6.2. Intracellular Adenosine Triphosphate (ATP) 

With some slight modifications, *E. faecalis* ATCC 29212 intracellular ATP concentration was determined using the method given by Sanchez et al. [20]. *E. faecalis* ATCC 29212 was resuspended in PBS to approximately 2 × 10^8^ CFU/mL concentration and then mixed with OEO at concentrations of 0 (control), 1/4 MIC, 1/2 MIC, and MIC, respectively. Then, the samples were cultured at 37 °C for 30 min. After incubation, cell samples were first lysed by ultrasound on ice, then centrifuged (5000× *g*, 5 min, 4 °C) and transferred supernatant into a 1.5 mL sterile tube and stored on ice. The concentrations of supernatant ATP were quantified using a microplate reader (Infinite^TM^ M200 PRO) after ATP assay (Beyotime Bioengineering Institute, Shanghai, China) was added to supernatant in white, opaque 96-well microtiter plates. The intracellular ATP concentration was estimated using a standard curve made up of luminescence values and ATP concentrations.

#### 2.6.3. Intracellular Reactive Oxygen Species (ROS)

With minimal adjustments, intracellular ROS levels were measured based on the description given by Jeon et al. [21]. The cultured bacterial suspension was diluted to 10^7^ CFU/mL, and OEO was added at different final concentrations (0, 1/4 MIC, 1/2 MIC, MIC) for 15 min or 30 min. To measure the ROS level in total, the OEO was removed and fluorescent probe 2′,7′-dichlorodihy-drofluorescein diacetate (DCFH-DA, Beyotime Institute of Biotechnology, Shanghai, China) was added. After incubation for 20 min, the bacteria cells were washed twice with PBS. Using fluorescence excitation and emission wavelengths of 488 and 525 nm, ROS generated in bacteria cells were detected. Finally, the data were normalized by survival bacteria number.

#### 2.6.4. Extracellular Malondialdehyde (MDA)

With minor changes, the MDA content was measured using an MDA test kit (Solarbio Life Sciences, Beijing, China) as described by Shao et al. [22]. The bacterial suspension was prepared according to 2.2 with a final concentration of 2 × 10^8^ CFU/mL. *E. faecalis* ATCC 29212 cultures were cultivated for 10 min at 37 °C with or without OEO (0 (control), 1/4 MIC, 1/2 MIC, MIC). The bacteria cells were centrifuged (8000× *g*, 10 min, 4 °C) after incubation and then stored on ice. The extract was added according to the assay kit’s instructions and the cultures were then incubated at 100 °C for 60 min. Finally, the amounts of MDA were measured at 450 nm, 532 nm, and 600 nm using a microplate reader (Infinite™ M200 PRO). 

MDA content was calculated according to this equation:

(1) $$MDA\;concent\;\left(nmol/mL\right)=5\times \left(12.9\times \left(\Delta A_{532\;\mathrm{nm}}-\Delta A_{600\;\mathrm{nm}}\right)-2.58\times \Delta A_{450\;\mathrm{nm}}\right)$$

#### 2.6.5. Bacterial Morphology 

According to Li et al. [23], the morphology of *E. faecalis* ATCC 29212 was observed using field emission scanning electron microscopy (FESEM). The prepared bacterial cultures were incubated at 37 °C for 1 h and 2 h with OEO at various concentrations (0 (control), 1/4 MIC, 1/2 MIC, MIC). The suspensions were centrifuged at 5000× *g* for 5 min after incubation and washed twice with PBS. After that, the bacteria were immobilized twice with 2.5% glutaraldehyde and then washed with PBS and sterile water after each immobilization. The bacteria were then dehydrated with aqueous ethanol at 30%, 50%, 70%, 80%, 90%, and 100% (*v*/*v*), followed by anhydrous ethanol. The samples were dried for 8–12 h in the fume hood. Finally, bacterial cells were observed using a FESEM (S-4800; Hitachi, Tokyo, Japan).

#### 2.6.6. Membrane Integrity 

Using a Flow Cytometer (CytoFLEX; Beckman, Shanghai, China), the influence of OEO on the membrane integrity of *E. faecalis* ATCC 29212 was examined based on the study conducted by Wu et al. [24]. The *E. faecalis* ATCC 29212 was treated by OEO with a series of MIC concentration gradients (0 (control), 1/4 MIC, 1/2 MIC, MIC). After incubation for 15 min at 37 °C, the cells were cleaned by centrifuging (10,000× *g*, 2 min, 4 °C) and resuspended with 0.85% NaCl (*m/v*). Then, the suspension was dyed for 15 min in the dark with propidium iodide (PI) and SYTO 9. The Flow Cytometric (FCM) analysis was performed after filtering through a 300-mesh screen.

### 2.7. Inactivation Effect of OEO against E. faecalis Biofilms

#### 2.7.1. Biofilm Formation

The *E. faecalis* ATCC 29212 was centrifuged (8000× *g*, 5 min, 4 °C) and resuspended in BHI broth to obtain a final bacterial concentration of 7.0 log CFU/mL. Then, the ultrasonic cleaned stainless-steel sheets (1 × 1 cm, grade 304, finish 4) were placed in a 24-well plate filled with 2 mL bacterial suspension and incubated at 37 °C for 24 h. 

#### 2.7.2. Viable Cell Enumeration

As described by Amalaradjou and Venkitanarayanan [25], OEO was tested for its ability to attack mature biofilms of *E. faecalis* on stainless-steel surfaces. To remove weakly adhering cells, following biofilm formation, the steel sheets were rinsed in sterile distilled water. The stainless-steel sheets were treated with solutions with or without OEO (0 (control), 2 MIC, 4 MIC, 8 MIC, 10 MIC, and 12 MIC) and incubated at 25 °C for 0, 1, 2, and 4 h. Lastly, the bacterial suspensions laid with glass beads (G8772, 425–600 µm; Sigma-Aldrich, St. Louis, MO, USA) were plated onto BHI plates and cultured for 24 h at 37 °C before counting.

#### 2.7.3. Confocal Laser Scanning Microscopy (CLSM) Observations

As indicated by Peng et al. [26], the effects of OEO on *E. faecalis* biofilm polysaccharides and structure were evaluated using CLSM (A1 confocal laser microscope; Nikon, Tokyo, Japan). Biofilms were produced on stainless-steel coupons (0.6 cm × 0.6 cm), as 2.7.1 described, before being treated with OEO at a concentration of 0, 4 MIC, 8 MIC, and 12 MIC at 25 °C for 1 h. The biofilms were next stained with concanavalin-A fluorescein conjugate (Con-A; Invitrogen/Molecular Probes, Eugene, OR, USA) at 4 °C for 30 min, immobilized by 2.5% glutaraldehyde for 2 h, then stained with Hoechst 33258 (Solarbio, Beijing, China) at room temperature for 20 min. Finally, using the CLSM, all images were captured with a 400× lens.

#### 2.7.4. Optical Microscope-Based Observation and Quantitation

The surface morphology of *E. faecalis* biofilms were observed utilizing an optical microscope (XTZ-D; Shanghai Optical Instrument Factory, Shanghai, China) as described in Li et al. [27]. After biofilm formation on the coverslips for 24 h in 24-well plates, the biofilm in each well was washed with PBS after the media was removed. At 25 °C for 1 h, the OEO was applied to each well to obtain final concentrations of 0 (control), 4 MIC, 8 MIC, and 12 MIC. After removal of OEO solutions, the coverslips were rinsed twice with PBS before being stained for 20 min with 0.4% (*w*/*v*) crystal violet. The coverslips were then rinsed with ultrapure water to eliminate any unbound crystal violet on the biofilm surfaces before being dried. The dyed biofilms were then examined under a 400× magnification optical microscope. On the digital image, using Image J software, the percentage of biofilm coverage was computed.

#### 2.7.5. Detection of the Viability of Cells in the Biofilm

MTT assay was used to test the viability of cells in *E. faecalis* biofilm as described by Parai et al. [28]. *E. faecalis* biofilms were formed by incubating a 96-well microtiter plate at 37 °C for 24 h. The OEO solutions were added to each well at a final concentration of 0 (control) MIC, 2 MIC, 4 MIC, 8 MIC, and 12 MIC and incubated at 25 °C for 1 h. Each well was cleaned twice with PBS. After that, 250 µL of 0.5 mg/mL 3-(4,5-dimethyl-2-thiazolyl)-2,5-diphenyl-2H-tetrazolium bromide (MTT; Beyotime Biotechnology, Shanghai, China) reagent solution was added and incubated. After removing the MTT solution, DMSO was added to dissolve the formazan crystals. Eventually, at 570 nm, the color changes caused by viable cell numbers were measured using microtiter plate reader (Infinite™ M200 PRO).

Percentage of viable cells was calculated according to this equation:

(2) $$Percentage\;of\;viable\;cells=\left[A_{treated}/A_{control}\;\right]\times 100$$

### 2.8. Application of OEO in Raw Chicken Breast Meat during Storage at 10 °C

#### 2.8.1. Antimicrobial Effect of OEO on Inoculated *E. faecalis* in Raw Chicken Breast Meat

Boneless, skinless chicken breasts were purchased from a local market (Yangling, China). Firstly, the chicken breasts were uniformly sliced (1 cm thick) and irradiated in a sterile sealed bag. After 2 s of 4 kGy irradiation, the background microorganisms on the raw chicken were fully eradicated, and it was subsequently stored in the refrigerator until usage at −20 °C. Briefly, chicken meat and 0.1% (*w*/*v*) buffered peptone water (BPW) were combined into a slurry with a meat-to-water weight ratio of 1:1. The bacterial suspension was prepared according to 2.2 and resuspended in 0.1% BPW to obtain a final concentration (~7.0 log CFU/mL). After inoculating the bacteria solution into the chicken meat samples, OEO was added into aseptic sealed bags to achieve final concentrations of 0, 10 MIC, 20 MIC, 30 MIC, and 40 MIC. Subsequently, the samples were kept in the refrigerator at 10 °C. The chicken samples were taken out at 0, 3, 6, 9, 12, 18, and 24 h, homogenized in 0.1% BPW for 3 min, and collected fluid (1 mL) serially diluted in PBS, plated onto BHI agar, and incubated for 24 h at 37 °C before enumeration.

#### 2.8.2. pH Assay

The pH was measured according to Zhao et al. [29]. A total of 5 g of the samples were homogenized completely for 120 s in 45 mL of distilled water, then allowed to stand for 30 min. A digital SMART SENSOR 838 pH-meter (Guangdong, China) was used to determine the pH of the homogenate.

### 2.9. Statistical Analysis

All the tests were done in triplicate, and the findings were presented as mean ± standard deviation. The statistical differences were assessed using SPSS version 19.0 (SPSS Inc., Chicago, IL, USA). Analysis of variance (ANOVA) procedure and Duncan’s post hoc test were used to determine statistically significant (*p* < 0.05) differences among treatments.

## 3. Results

### 3.1. MICs and MBCs

The MICs of OEO against *E. faecalis* strains ranged from 0.25 to 0.50 μL/mL, as presented in Table 1. At 0.25 µL/mL, OEO reduced the growth of ATCC 29212, SX20FC319 and SX20FCN9 and had bactericidal activity at 0.50 µL/mL. OEO inhibited the growth of SX20FC004, and SX20FC318 at 0.50 µL/mL and had bactericidal effects at 1.00 µL/mL. For SX20FC006, SX20FC181, SX20FC182, SX20FCN4, and SX20FCN6, the MICs and MBCs of OEO were found to be 0.50 µL/mL. 

### 3.2. Growth Curves

**Figure 1:**
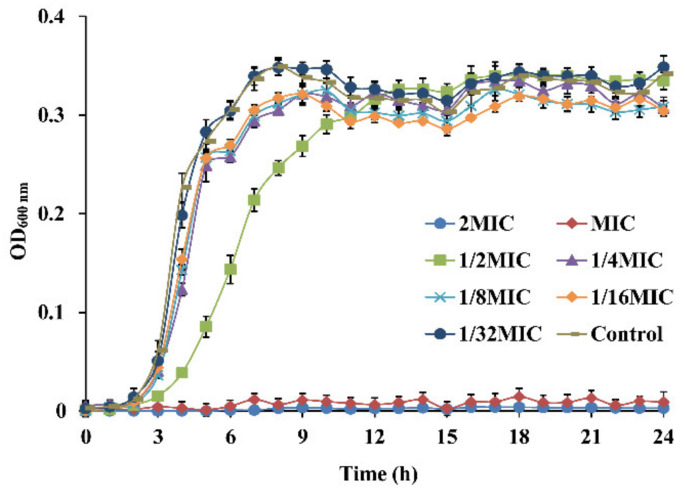
*E. faecalis* ATCC 29212 growth curves in BHI broth with different concentrations of OEO.

Figure 1 shows the influence of OEO at 1/32 MIC to 2 MIC on the growth of *E. faecalis* ATCC 29212. Results showed that OEO completely suppressed *E. faecalis* growth at MIC and 2MIC. Furthermore, in the presence of 1/2 MIC OEO, the lag phase of *E. faecalis* was protracted compared to the control, and the growth rate was slowed. Furthermore, *E. faecalis* grew similarly to the control in the presence of OEO at 1/32 MIC, 1/16 MIC, 1/8 MIC, and 1/4 MIC, but these concentrations still suppressed growth.

### 3.3. Antimicrobial Effect of OEO on E. faecalis

**Figure 2:**
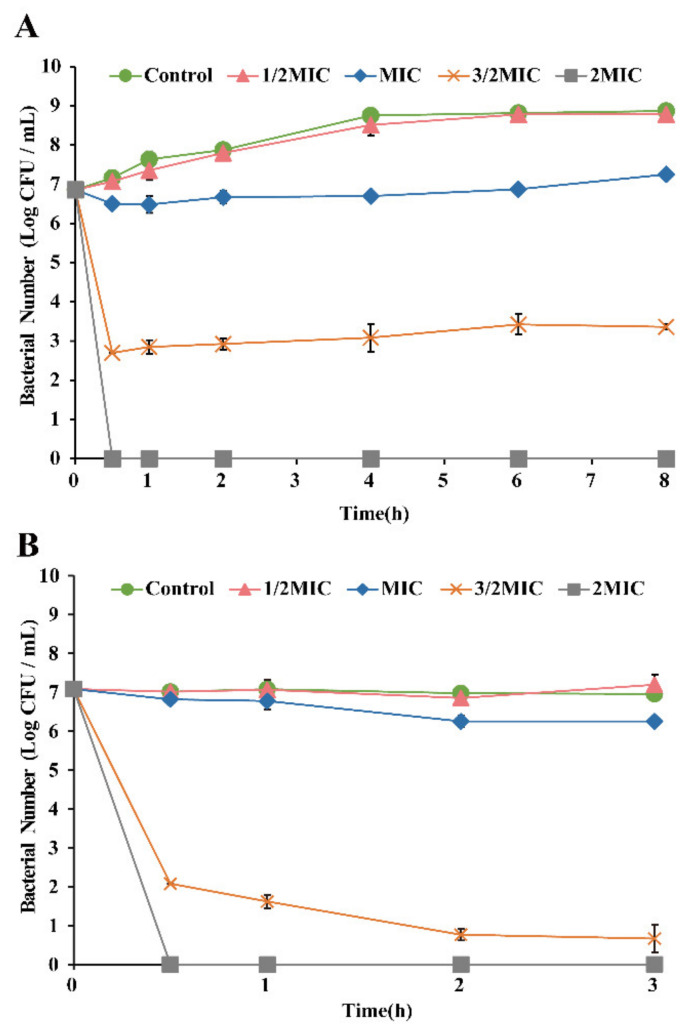
Effect of OEO on *E. faecalis* ATCC 29212 populations in BHI broth (**A**) and PBS (**B**).

Figure 2A shows changes in the number of *E. faecalis* in BHI broth treated with OEO at 37 °C. The initial number of *E. faecalis* in BHI broth was approximately 6.9 log CFU/mL. After the untreated strain grew in BHI broth for 8 h, the *E. faecalis* increased by 2.0 log CFU/mL, and the *E. faecalis* increased by 1.9 and 0.4 log CFU/mL at the concentrations of 1/2 MIC and MIC, respectively. At the concentration of 3/2 MIC, the *E. faecalis* decreased by 3.5 log CFU/mL. In the meantime, when *E. faecalis* was treated with OEO at 2 MIC for 30 min, the number of bacteria had decreased to the point (3.33 CFU/mL) that it was no longer detectable.

The initial number of *E. faecalis* in PBS was approximately 7.1 log CFU/mL (Figure 2B). After the untreated strains grew in PBS for 3 h, the *E. faecalis* decreased by 0.13 log CFU/mL, with little change. There was also no significant change in 1/2 MIC treatment. In MIC concentration and 3/2 MIC concentration, the *E. faecalis* decreased by 0.8 and 6.4 log CFU/mL, respectively. In the meantime, when *E. faecalis* was treated with OEO at 2 MIC for 30 min, the number of bacteria dropped below the detection limit (3.33 CFU/mL).

### 3.4. Antibacterial Mechanism of OEO against E. faecalis

#### 3.4.1. Membrane Potential

**Figure 3:**
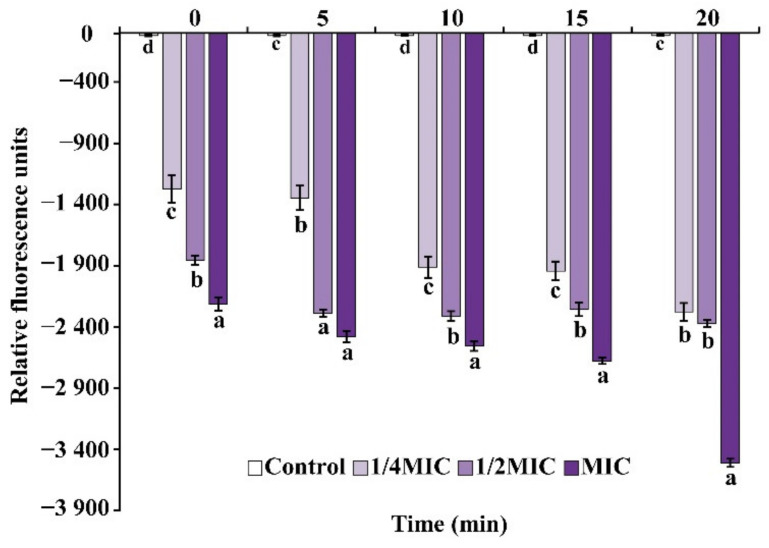
Effect of OEO on the membrane potential of *E. faecalis* ATCC 29212. Different small letters in each time indicate significant (*p* < 0.05) statistical differences among treatments.

The cell membrane potential of *E. faecalis* treated with OEO showed hyperpolarization, which was concentration-dependent and time-dependent. In comparison with the control, cells’ exposure to OEO exhibited rapid cell membrane hyperpolarization as evidenced by an increase in fluorescence (negative values), which was shown in Figure 3. Furthermore, as OEO concentration increased from 1/4 MIC to MIC, the degree of hyperpolarization increased.

#### 3.4.2. Intracellular ATP 

**Figure 4:**
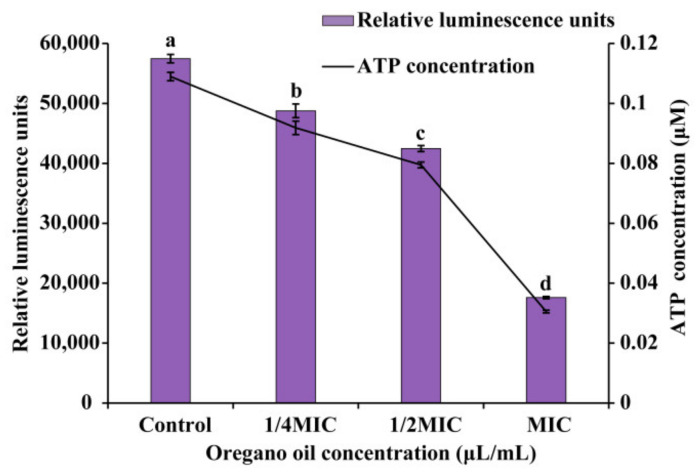
Effects of OEO on intracellular adenosine triphosphate (ATP) of *E. faecalis*. Different lowercase letters indicate statistically significant differences between the means (*p* < 0.05).

OEO could cause *E. faecalis* ATCC 29212 cells to release intracellular ATP (Figure 4). The relationship between relative luminescence units and ATP concentration was well-defined (y = 508,386x + 2070; R^2^ = 0.9999). As demonstrated in Figure 4, the intracellular ATP concentrations of *E. faecalis* exposure to OEO at 1/4 MIC,1/2 MIC and MIC dropped significantly (*p* < 0.05) in a dose-dependent manner when compared to the control. The control had an intracellular ATP content of 0.11 ± 0.0014 μmol/L, which fell to 0.09 ± 0.0023 μmol/L and 0.08 ± 0.0010 μmol/L following 30 min of treatment with OEO at 1/4 MIC and 1/2 MIC, respectively. In *E. faecalis* cells treated with OEO at MIC for 30 min, the ATP concentration was lowered to 0.03 ± 0.0005 μmol/L. Furthermore, a significant (*p* < 0.05) difference was found in intracellular ATP concentrations corresponding to the three concentrations of OEO.

#### 3.4.3. Intracellular ROS 

**Figure 5:**
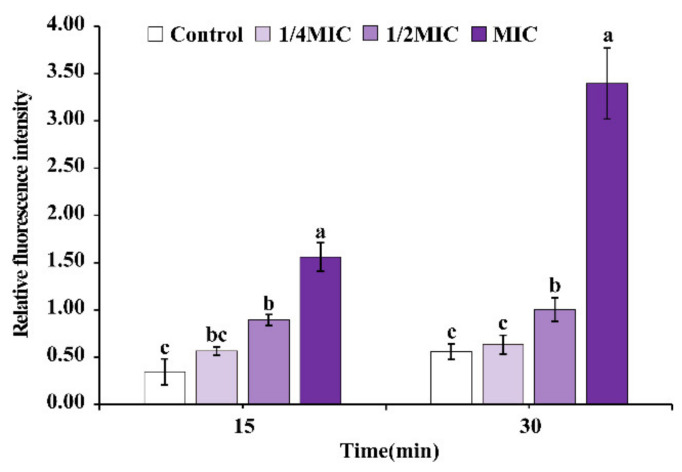
Effect of OEO treatment for 15 and 30 min on intracellular reactive oxygen species (ROS) levels in *E. faecalis*. Different small letters in each time indicate significant (*p* < 0.05) statistical differences among treatments.

As shown in Figure 5, OEO increased intracellular ROS levels in *E. faecalis* ATCC 29212. After being treated with different concentrations of OEO for 15 min, 1/2MIC OEO, and MIC OEO treatment samples resulted in a significantly (*p* < 0.05) increased generation of intracellular ROS compared to untreated control. Similarly, the intracellular ROS level was likewise elevated when the treatment period was 30 min. Moreover, the level of intracellular ROS generation was on the rise as treatment time of OEO increased, and ROS generation reached the highest level when *E. faecalis* was treated with MIC OEO for 30 min.

#### 3.4.4. Extracellular MDA

**Figure 6:**
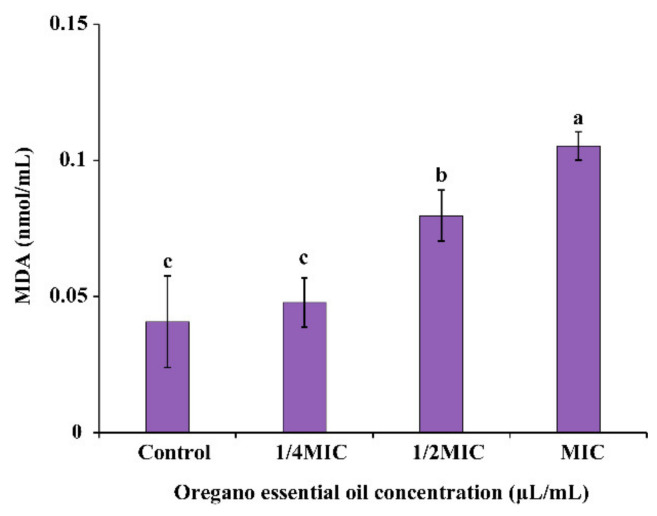
Extracellular malondialdehyde (MDA) in *E. faecalis* ATCC 29212 treated with OEO. Different lowercase letters indicate statistically significant differences between the means (*p* < 0.05).

As demonstrated in Figure 6, comparing the treatment with OEO and the control samples, the extracellular MDA level of *E. faecalis* decreased concentration-dependently. The content of extracellular MDA rose considerably from 0.041 ± 0.017 nmol/mL to 0.079 ± 0.009 nmol/mL (*p* < 0.05) and 0.105 ± 0.005 nmol/mL (*p* < 0.05) when *E. faecalis* was treated with OEO at 1/2 MIC, and MIC concentrations increased significantly.

#### 3.4.5. Membrane Integrity

**Figure 7:**
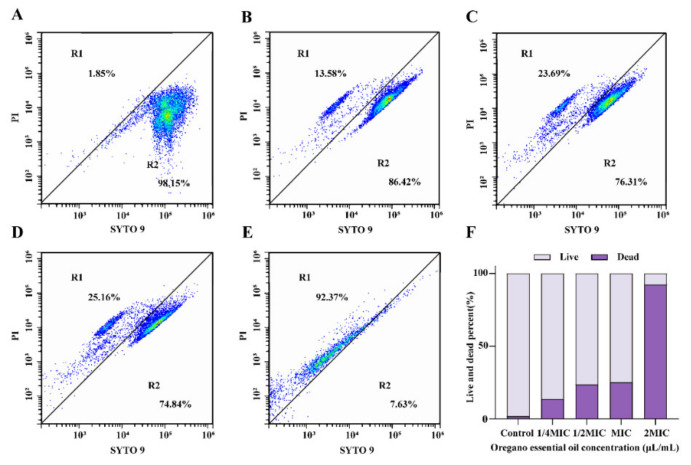
Flow cytometry observations of OEO’s effects on the cell membrane integrity of *E. faecalis* ATCC 29212. (**A**) Untreated. (**B**) Treated with OEO at 1/4 MIC. (**C**) treated with OEO at 1/2 MIC (**D**) treated with OEO at MIC. (**E**) treated with OEO at 2 MIC. (**F**) Live and dead cell percentage. Cells were stained with SYTO9 and PI simultaneously. R1: dead or membrane-damaged cells. R2: living cells.

FCM was used to determine the loss of membrane integrity in *E. faecalis* treated with OEO by detecting the fluorescent signals from SYTO 9 and PI. As a result, in the dot plots, bacterial cell populations were concentrated in two discrete blocked regions: R1 and R2. R1 correlates to high blue fluorescence, indicating dead or membrane-damaged cells, and R2 correlates to strong red fluorescence, indicating living cells. After exposure to MIC of OEO (Figure 7D), the percentage of cells with dead or membrane-damaged increased from 1.85% to 25.16% compared with the negative control (Figure 7A). After exposure to 2 MIC of OEO (Figure 7E), the percentage of cells with dead or membrane-damaged increased to 92.37%. The results demonstrated that OEO exposure can induce damage in the cell membrane of *E. faecalis* by a loss of membrane integrity.

#### 3.4.6. Cell Morphology

**Figure 8:**
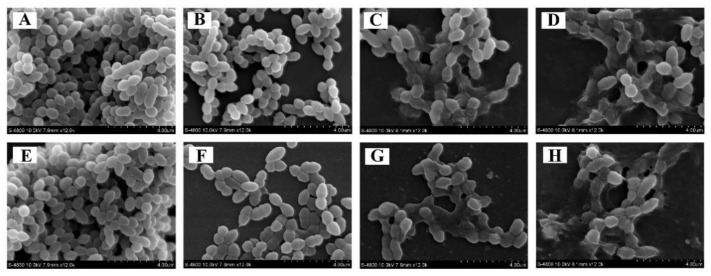
Observations of *E. faecalis* ATCC 29212 using field emission scanning electron microscopy (×12,000 magnification). Untreated for 1 (**A**) and 2 h (**E**). Treated with OEO at 1/4 MIC for 1 (**B**) and 2 h (**F**). Treated with OEO at 1/2 MIC for 1 (**C**) and 2 h (**G**). Treated with OEO at MIC for 1 (**D**) and 2 h (**H**).

Untreated *E. faecalis* ATCC 29212 has a typical elliptical form with a smooth appearance (Figure 8A,E). *E. faecalis* cells exhibited an irregular outer surface, were not uniform in size and distribution, and tended to each other when exposed to OEO at different concentrations (Figure 8B–D,F–H). Moreover, the cells treated with MIC OEO had hazy surfaces, with fragmentation, aggregation, and adhesion of massive surface collapse of injured cells or cellular debris (Figure 8D,H). Furthermore, as treatment time went on, the level of morphological damage grew. 

### 3.5. Inactivation of OEO on E. faecalis in Biofilms 

#### 3.5.1. Viable Cell Enumeration

**Figure 9:**
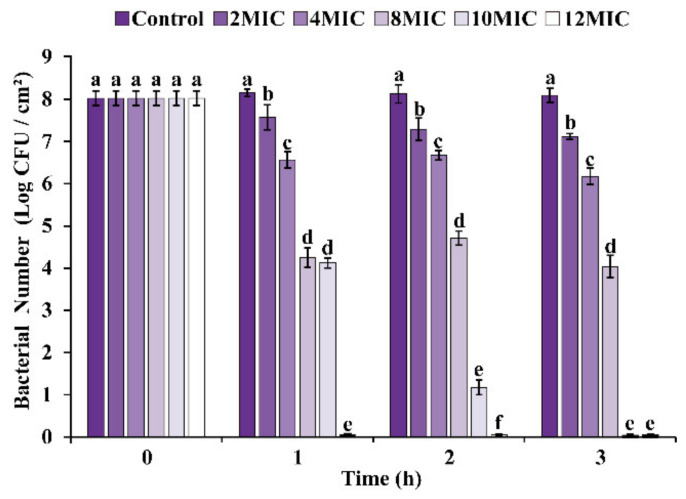
Effects of OEO on the number of viable cells of *E. faecalis* ATCC 29212 biofilm. Different small letters in each time indicate significant (*p* < 0.05) statistical differences among treatments.

Figure 9 depicts changes in viable biofilm cells produced on stainless-steel sheets treated with OEO at 25 °C. All treatment groups had the same number (~8.0 log CFU/cm^2^) of viable *E. faecalis* cells on stainless-steel surfaces at the beginning of treatment. Untreated biofilms maintained a cell population of about 8.1 log CFU/cm^2^ throughout the experiment, while viable cell counts decreased to 6.9, 5.7, and 3.4 log CFU/cm^2^ after exposure to OEO at 2 MIC, 4 MIC, and 8 MIC for 4 h, respectively. In addition, after 3 h and 1 h treatment, cells that are viable in biofilms exposed to 10 MIC and 12 MIC OEO were below detectable levels.

#### 3.5.2. Biofilm Morphology

**Figure 10:**
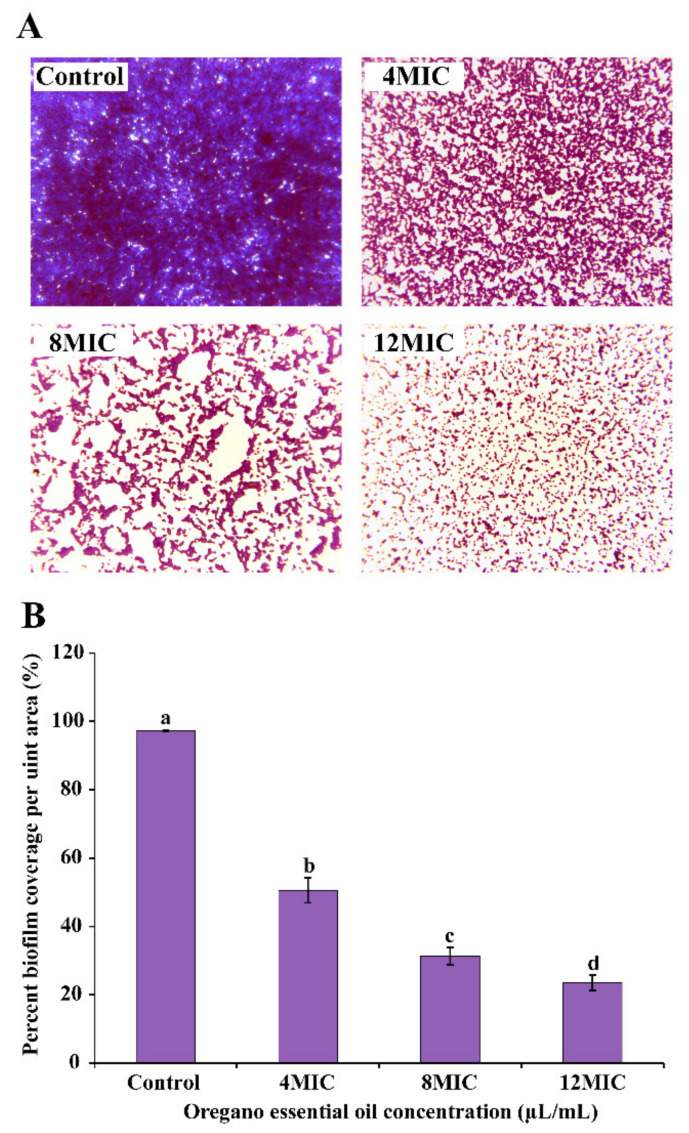
*E. faecalis* ATCC 29212 biofilms treated with varying doses of OEO as seen under a light microscope (**A**). Optical Microscope-Based Observation was used to calculate the percent biofilm coverage per unit area (**B**). Different lowercase letters indicate statistically significant differences between the means (*p* < 0.05).

After crystal violet staining, at 400× magnification, *E. faecalis* ATCC 29212 biofilms with and without OEO treatment were examined (Figure 10). The biofilm of *E. faecalis* that had not been treated was complete, consistently distributed, and covered the full field of vision (Figure 10A). Following OEO treatment, however, a significant (*p* < 0.05) decrease in biofilm biomass was observed. In response to increased OEO concentration, biofilm dispersal was seen to increase. In addition, the percent biofilm coverage determined from light microscope pictures followed the same pattern as the specific biofilm formation. Following OEO treatment at 4 MIC, 8 MIC, and 12 MIC, the percent biofilm coverage of *E. faecalis* ATCC 29212 was decreased by 46.60%, 65.86%, and 73.64%, respectively (Figure 10B).

#### 3.5.3. CLSM Observations

**Figure 11:**
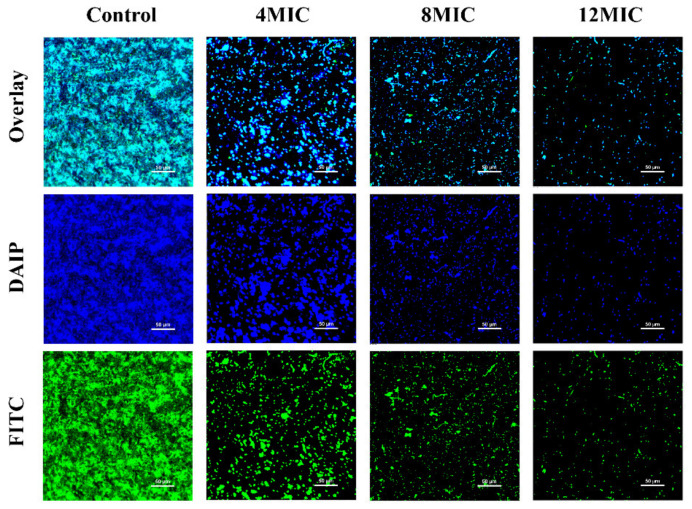
CLSM observations (400× magnification) of OEO on the *E. faecalis* ATCC 29212 biofilm.

The effects of OEO on *E. faecalis* biofilms are shown in Figure 11. The bacteria from *E. faecalis* that had not been treated were intimately linked, generating a high number of micro-colonies. The polysaccharides are uniformly distributed on the biofilm and aggregate into polymer blocks. However, following OEO treatment, the green fluorescence and blue fluorescence were reduced. The biofilm was dispersed, and the polysaccharide content was dramatically reduced, according to these findings.

#### 3.5.4. Effect of OEO on Cell Viability in Biofilm

**Figure 12:**
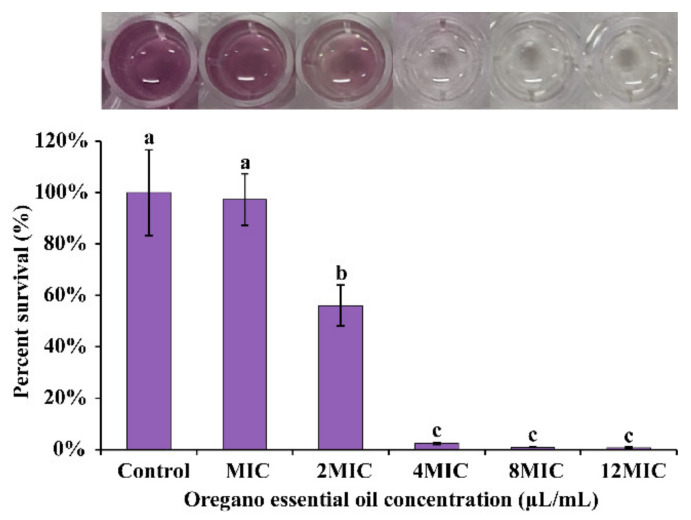
Effects of OEO on biofilm reduction and cell viability of *E. faecalis* ATCC 29212. Different lowercase letters indicate statistically significant differences between the means (*p* < 0.05).

The MTT assay was used to further establish OEO’s ability to inhibit *E. faecalis* metabolic activity. As demonstrated in Figure 12, in addition to MIC OEO treated group, other treated samples had a significant (*p* < 0.05) drop in cell-viable percentage when compared to the control samples (100%). The percentage measured at the MIC, 2 MIC, 4 MIC, 8 MIC, and 12 MIC were 97.309%, 56.049%, 2.381%, 0.912%, and 0.707%, respectively.

### 3.6. Application of the OEO to Raw Chicken Breast Meat

#### 3.6.1. Antimicrobial Activity

**Figure 13:**
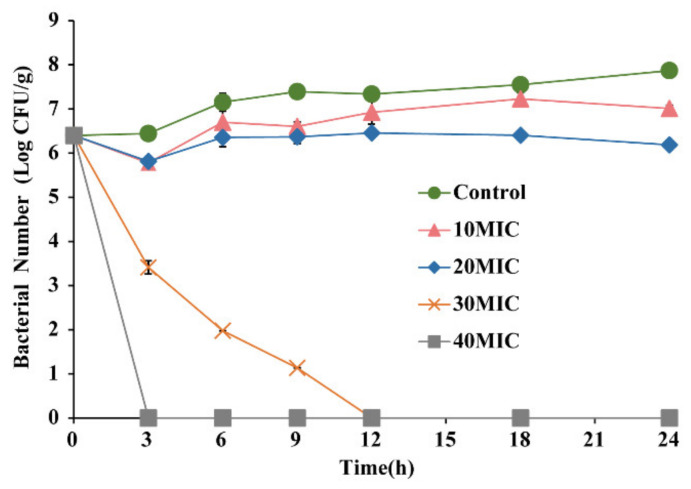
Antimicrobial activity of OEO on *E. faecalis* ATCC 29212 in raw chicken.

Figure 13 depicts the antibacterial activity of OEO against *E. faecalis* in raw chicken. The total number of *E. faecalis* cells in all raw chicken samples was 6.4 log CFU/g at 0 h post-inoculation. Within 24 h, the control group’s bacterial count increased from 6.40 ± 0.15 log CFU/g to 7.87 ± 0.18 log CFU/g, while the 10 MIC and 20 MIC group’s bacterial counts fell by 0.9 and 1.7 log CFU/g, respectively. In addition, after 3 h and 12 h treatment, the bacterial numbers in the 40 MIC and 30 MIC groups were below detectable levels. 

#### 3.6.2. pH Value

Table 2 shows the results of the pH values of chicken meat treated with OEO. As can be seen, variations in pH values of different treatments followed the same pattern, with values increasing at first and subsequently decreasing. The pH value of treated raw chicken at 0 and 10 MIC concentration OEO was approximately the same at each time point. Except for 30 MIC and 40 MIC, the pH of treated meat increased significantly (*p* < 0.05) towards the end of the storage.

## 4. Discussion

OEO was found to have inhibitory activity against ten strains of *E. faecalis*, with the MIC values from 0.25 to 0.50 μL/mL (235 to 470 μg/mL) (Table 1) in this investigation. Other natural compounds’ inhibitory effects on *E. faecalis* have also been investigated in previous investigations. The MIC of thyme oil against *E. faecalis* was determined to be 512 μg/mL by Liu et al. [30]. In addition, Zhou et al. [1] reported that the MIC of *Perilla frutescens* essential oil against *E. faecalis* was 0.50 μL/mL. Silva et al. [31] showed that the MICs of *Rosmarinus officinalis*, *Zingiber officinale,* and *Citrus aurantium bergamia* against *E. faecalis* were 114.87, 219.50, and 219.25 mg/mL. Therefore, in comparison with these natural substances that have been reported so far, the antimicrobial activity of OEO against *E. faecalis* was higher. 

Membrane potential is one of the most significant parameters of the bacterial function, as it is linked to antimicrobial agent uptake and bactericidal activity [32]. In this study, OEO caused hyperpolarization of the *E. faecalis* cell membrane (Figure 3). In previous studies, *trans*-cinnamaldehyde could cause cell membrane hyperpolarization as its antibacterial mechanism [33]. Similarly, carvacrol, eugenol, and citral could cause cell membrane hyperpolarization in *L. monocytogenes* [34]. Membrane damage can occur as a result of depolarization or hyperpolarization. Previous research has found that cell membrane hyperpolarization is linked to pH change and external K^+^ diffusion, while membrane depolarization is mostly caused by the opening of the Na^+^ channel, which permits Na^+^ ions to permeate into cells [20,35].

In bacteria, ATP is one of the most important energy-related molecules, and it is very important for microbial cell activity, growth, and survival [36,37]. Intracellular ATP content is a key index of available energy in microorganisms. When compared to the control group, OEO significantly (*p* < 0.05) decreased the intracellular levels of *E. faecalis* (Figure 4). In the study of Zhang et al. [38], the intracellular ATP concentration of *L. monocytogenes* treated with eugenol decreased significantly (*p* < 0.05). Cui et al. [39] found that when *Helichrysum italicum* oil was used to treat *S. aureus*, intracellular ATP concentration was considerably (*p* < 0.05) lowered. Recent research has concluded that the observed drop in intracellular ATP content could be attributable to fast ATP hydrolysis or intracellular ATP leakage through the cell membrane due to changes in cell membrane permeability [20,35].

ROS is an important signal leading to cell damage, which plays an important role in understanding the target of natural products on pathogenic bacteria. OEO considerably raised the level of intracellular ROS in *E. faecalis* in this investigation (Figure 5). In previous reports, Shi et al. [40] found that the ROS formation in *E. coli* and *S. aureus* was remarkably increased with the concentration of Octyl gallate. Similarly, Basil Essential Oil was reported to increase the production of ROS in *L. monocytogenes* [41]. In addition, ROS can induce intracellular damage such as aberrant protein oxidation, DNA damage, and lipid peroxidation, all of which reduce cell viability [42].

High levels of ROS in organisms attack cell membranes and cause peroxidation [43]. MDA is one of the main products of membrane lipid peroxidation and a useful indication for determining how much membrane lipid peroxidation has occurred. The increase of the extracellular MDA content of *E. faecalis* treated with OEO was found to be compatible with the rising trend of ROS in this investigation (Figure 6). Similarly, Montanari et al. [44] reported that *Schinus terebinthifolius* leaves oil could increase the content of MDA of *E. coli* and *S. aureus*. In addition, Lee and Lee [45] found that resveratrol treatment could increase the concentration of MDA of *S. typhimurium* from 0.62 to 1.45 nM. In this study, excessive ROS may have triggered lipid peroxidation, which resulted in a rise in MDA in *E. faecalis*. Lipid peroxidation is a chain reaction caused by the addition of oxygen free radicals, resulting in oxidative damage of polyunsaturated fatty acids. The cell membrane composed of polyunsaturated fatty acids is the main target of ROS attack. Therefore, ROS attacks can lead to severe membrane damage [44].

With the co-staining of PI and SYTO 9, the effect of OEO on the cell membrane integrity of *E. faecalis* was observed by FCM. This study confirmed that the percentage of cells that were dead or had membrane damage increased significantly after OEO treatment (Figure 7). Similarly, Wu et al. [24] used the same method to demonstrate that 3-*p*-*trans*-Coumaroyl-2-hydroxyquinic acid treatment impaired membrane integrity of *S. aureus*. Tian et al. [46] found that the cell membrane of *Enterobacter sakazakii* was destroyed by thymol treatment. Combined with the experimental results of MDA and ROS in this study, we suspected that the damage to membrane integrity of *E. faecalis* was due to the accumulation of ROS and membrane lipid peroxidation caused by OEO treatment.

The FESEM analysis of this study showed that the morphology of *E. faecalis* was altered by OEO (Figure 8). After exposure to OEO, the cell surface contracted, stuck, and collapsed. In previous reports, Sun et al. [47] observed by scanning electron microscopy (SEM) that membrane shape, aggregation, and leaking of cellular contents were seen in *St. aureus*, *S. enteritidis*, *L. monocytogenes,* and *V. parahaemolyticus* treated with anthocyanins at the MBC concentration. Similarly, Becerril et al. [48] observed by SEM that the appearance of *E. coli* cells changed irregularly when treated with lauryl arginine ethyl ester for 5 min, with obvious pore formation on the surface, serious shrinkage, and rupture of bacteria. Furthermore, changes in *E. faecalis* cell morphology may have been caused by OEO-induced membrane permeability and integrity damage, according to the results of ATP, membrane potential, ROS and MDA assays.

Bacterial biofilm consists of cell groups embedded in extracellular polymers, which protect bacteria from adverse environmental conditions, such as disinfection, antibiotic treatment, etc. [49]. OEO effectively reduced the number of viable cells in an *E. faecalis* biofilm grown on a stainless-steel surface in this investigation (Figure 9). Kang et al. [50] studied peppermint essential oil’s anti-biofilm effectiveness against mature *S. aureus* biofilm and found that a high concentration of peppermint essential oil (≥4 mg/mL) could lower the number of viable cells in *S. aureus* biofilm below the detection threshold. Kang et al. [51] confirmed that 2 mg/mL gallic acid treatment reduced the viable cells of *Shigella flexneri* biofilm by about 4 log CFU/mL.

After crystal violet staining, the effect of OEO treatment on the morphology of *E. faecalis* biofilms was observed using a light microscope. Following OEO treatment, biofilm biomass decreases significantly, with increasing dispersal observed as OEO concentration rises (Figure 10). Bai et al. [52] found that a remarkable reduction in *S. aureus* biofilm was observed in shikimic acid (SA)-treated slides using the same method, and the biofilm biomass in the presence of SA was significantly reduced in a dose-dependent manner. On the glass slides, Yang et al. [53] found that biofilms of CoQ0-treated *S.* Typhimurium were significantly reduced.

Con-A and Hoechst 33258 were used in the CLSM study. Con-A stains the sugar residues in biofilm polysaccharides green, while Hoechst 33258 stains the bacterial cells blue. CLSM-analysis revealed that, compared with the control group, the structure of biofilm was dispersed and the abundance of polysaccharides was decreased after the treatment of OEO (Figure 11). Polysaccharides, together with extracellular DNA and proteins, form extracellular polymers (EPS) as a matrix that effectively binds the microbial cells [54]. Guo et al. [55] confirmed that CoQ0 treatment could damage the structure of *Cronobacter sakazakii* biofilm. Liu et al. [56] confirmed that 2.5 mg/mL phenyllactic acid treatment significantly reduced the EPS content in the biofilm of *E. faecalis* compared with the control group (*p* < 0.05). 

The effect of OEO on *E. faecalis* biofilm metabolic activity was estimated by the MTT assay. This study showed that after OEO treatment, compared with the control group, the metabolic activity of *E. faecalis* biofilm was decreased (Figure 12), and this result matches the results of the viable cell enumeration assay for biofilms treated with OEO. Costa et al. [57] reported that biofilm metabolic activity of *S. aureus* was reduced by the antimicrobial peptide P34. Trevisan et al. [58] confirmed that carvacrol at 2 MIC and 4 MIC decreased *S.* Typhimurium biofilm activity compared with the control (*p* < 0.05). In addition, the results of the antibacterial mechanism assay showed that OEO can inactivate *E. faecalis* by impairing the membrane permeability and integrity of the cells. Thus, the lethal action of OEO on *E. faecalis* in mature biofilms is achieved mostly by disrupting the cell membrane of the biofilm cells.

*E. faecalis* is a common contaminant of many food products, including meat, dairy products, and ready-to-eat salads [59]. In this study, OEO can effectively inhibit *E. faecalis* in raw chicken breast (Figure 13). Yang et al. [60] found that CoQ0 with 20 MIC concentration can reduce *S.* Typhimurium in the chicken breast by about 1.4 log CFU/mL within 9 h. Luo et al. [61] studied the ability of OEO to inactivate *Vibrio vulnificus* at initial concentrations of 6.56 log CFU/g in fresh oysters and found that 0.09% (*v*/*v*) of OEO treatment could reduce the bacterial count to 3.39 log CFU/g at 10 h. However, when comparing the antibacterial effects of OEO in BHI broth and chicken breast surimi (Figure 2 and Figure 13), it was shown that OEO in chicken breast surimi had a lesser antibacterial impact. We suspect that the possible reason is that the temperature of OEO treatment is different (BHI broth: 37 °C; chicken breast: 10 °C); the protein in chicken breast reduced the effective contact area between OEO and *E. faecalis*. 

The physicochemical characteristics are regarded as one of the most important factors for consumers in determining meat quality and meat freshness [62]. The rise in pH was linked to the proliferation of spoilage bacteria capable of degrading proteins and producing volatile bases [63]. In this study, the pH values of raw chicken breast samples treated with 20 MIC, 30 MIC, and 40 MIC OEO decreased when compared to control samples, which could be connected to OEO’s antibacterial action.

## 5. Conclusions

In summary, our research found that OEO exerted an inactivation effect on *E. faecalis* by inducing the rise of ROS levels to cause the lipid peroxidation of the cell membrane, destroy the membrane’s permeability and integrity, and then change the bacterial shape. The cell membrane hyperpolarized, intracellular ATP concentrations dropped and extracellular MDA levels rose, further confirming the membrane damage. In addition, *E. faecalis* was also effectively inactivated by OEO in mature biofilm on stainless-steel, decreasing the *E. faecalis* biofilm metabolic activity and dispersing the biofilm structure. Moreover, OEO showed antibacterial activity against *E. faecalis* in raw chicken breast; as the concentration increases of OEO, the rate of increase in pH values decreased. These findings show that OEO could be utilized to successfully manage *E. faecalis* in food production and processing contexts.

## Figures and Tables

**Table 1:** The MICs and MBCs of OEO against *E. faecalis*.

Strain	Origin	MIC(μL/mL)	MBC(μL/mL)
ATCC 29212	Urine	0.25	0.50
SX20FC004	Chicken cloacal swab	0.50	1.00
SX20FC006	Chicken cloacal swab	0.50	0.50
SX20FC181	Pig nasal swab	0.50	0.50
SX20FC182	Pig nasal swab	0.50	0.50
SX20FC318	Bovine breast swab	0.50	1.00
SX20FC319	Bovine breast swab	0.25	0.50
SX20FCN4	Raw milk	0.50	0.50
SX20FCN6	Raw milk	0.50	0.50
SX20FCN9	Raw milk	0.25	0.50

**Table 2:** pH values of raw chicken breast meat with OEO during storage at 10 °C.

Storage Time (Hour)	0	3	12	24
Control	6.15 ± 0.01 ^cC^	6.18 ± 0.03 ^cC^	6.35 ± 0.01 ^abA^	6.31 ± 0.01 ^aB^
10 MIC	6.18 ± 0.01 ^bD^	6.21 ± 0.01b ^cC^	6.33 ± 0.01 ^bA^	6.32 ± 0.01 ^aB^
20 MIC	6.20 ± 0.01a ^bC^	6.28 ± 0.01 ^aA^	6.27 ± 0.01 ^cA^	6.23 ± 0.01 ^bB^
30 MIC	6.21 ± 0.01a ^bC^	6.26 ± 0.02 ^aB^	6.36 ± 0.02 ^aA^	6.23 ± 0.01 ^bC^
40 MIC	6.21 ± 0.02 ^aB^	6.22 ± 0.01 ^bB^	6.33 ± 0.02 ^bA^	6.21 ± 0.01 ^cB^

Values are means ± SD from triplicate determinations. Different lowercase letters show significant variations as a result of the meats being treated with different concentrations of OEO (*p* < 0.05), whereas different uppercase letters indicate significant differences as a result of the storage duration (*p* < 0.05).

## Data Availability

The data presented in this study are available on request from the corresponding author.
